# Popular Fallacies about Cancer

**Published:** 1888-05-12

**Authors:** 


### The Hospital

A Weekly Institutional Journal of

**Science, Medicine, Nursing, and Philanthropy.**

*For Hospitals, Asylums, Medical Practitioners, Students, Nurses, Families, Parishes, Congregations, and the Charitable Public.*

Vol. IV. No. 85.] [May 12, 1888.

### Popular Fallacies about Cancer.

In large towns where much interest is taken by people generally in the diseases from which they and their friends suffer and die, cancer occupies a very unenviable degree of prominence and nortoriety. To men it is a name which causes unpleasant sensations; to women it is a word of terror. The disease is in itself often painful in a high degree; not seldom it is loathsome in its form and progress; and in very many cases its commencement is the first stroke of doom. Two circumstances are present with this generation which, whilst they tend to its advantage, do not favour its peace of mind. These are a great increase of knowledge and a corresponding development of sensitiveness. In London the knowledge of medicine, as a science and an art, possessed by persons of average education, is tenfold greater than that possessed by people of the same class in country districts, and more than ten times greater than that possessed by their metropolitan ancestors a hundred years ago. In these times there are many fathers and mothers of large families whose acquaintance with the family and other physicians has been so extensive that they have a more rational understanding of medical science and practice than the average medical practitioner of some countries. To such people the mention of a disease like consumption or cancer in connexion with any member of their families causes a thrill of the most painful dread. The very names of these diseases strike terror to the sensitive mind.

A little knowledge, though it be a very useful thing, undoubtedly has its disadvantages. The man of experience passes through the churchyard at midnight unmoved; the child or the timid woman sees ghosts. It is exactly so in the case of certain derangements to which man is subject. The medical man knows that the chances in his favour are as a hundred or a thousand or ten thousand to one, and he comforts himself with this assurance. The nervous woman who hears that one neighbour has cancer of the breast, another cancer of the liver, and that a distant relation is dying of the disease in some other organ, at once concludes that cancer is becoming terribly, alarmingly frequent, and that she herself is more likely than otherwise to fall a victim to its dreadful power. In such circumstances, it is well worth while to banish a few of the ghosts which haunt the imagination by the publication of certain facts.

Is cancer hereditary? What an anxious question to some mothers of families! Their own mothers died of the disease. Are they, too, going to die of it, and to leave their children just when a mother's care is most needed? And will their daughters, now so happy and unconscious of impending danger, be liable also to share in such a dread inheritance? These and similar questions add many a heartache to the other cares of a gentle mother's life. The present belief of the medical profession is one which should give comfort to all anxious minds. Let no one labour to diminish the comfort by fastening on the words "present belief." The scientific chiefs of medicine in these times are men to be trusted, men to be proud of. Not a few of them belong to our own country. More than twenty-five years ago Mr. Jonathan Hutchinson, "whose opinions," in the words of an eminent scientist, "carry the greatest weight," expressed his disbelief in hereditary origin as an effective cause of cancer. With twenty-five years of added experience of the most varied and extensive kind, Mr. Hutchinson has seen no reason to change his views. Last year he publicly stated, before a medical audience of the first eminence, the conviction that "It is utterly useless to employ such a term as hereditary transmission of cancer in such a sense as we speak of the transmission of some other diseases." "Within the past few years a large number of the most eminent pathologists have become adherents of the doctrine that cancer is primarily a local disease, and that the constitutional affection is a secondary result." It may be taken for granted, therefore, that in the proper sense of the word, cancer is not hereditary. There may be in certain families tendencies which favour the development of such diseases as cancer; but these tendencies may be modified or "grown out of." The point to remember, and to remember with confidence and hope, is that, according to the best medical authorities, cancer is not hereditary.

Is a tumour a cancer? A class of medical students would smile at such a question. But to many people the word tumour imports all the terrors which attach to the name of cancer. Tumour is a most harmless word, and yet the considerate physician never employs it to his patient if he can help it. He knows that if he should do so an amount of suffering and anxiety might result which he could hardly calculate. "The doctor says I have a tumour!" cries Mrs. Smith. Thereupon her imagination takes the bit between its teeth, and no Mazeppa on untamed steed ever rushed off on a more unrestrained career. If the doctor says Mrs. Smith has a tumour, Mrs. Smith is convinced that he means she has a cancer, and merely uses the word tumour because he does not wish to frighten her. Nothing can be further from the truth. A "tumour" is a swelling—a tumid or swollen condition; nothing more. Nobody is alarmed by the word "swelling;"

why should they be frightened when it is translated into the Latin tongue and called a "tumour"? An ordinary knee-joint may be called a tumour, inasmuch as it is a more prominent part than the portion of the leg or thigh next to it. An enlarged tonsil is a tumour; a wart is a tumour; a swollen gland is a tumour; a puffed-out black eye is a tumour; any swelling of any kind, whether normal or abnormal, may properly enough be called a tumour. The "tumour" has long been a dreadful ghost; let him in future be nothing more than a straw-stuffed man, and let his proper designation be not "ghost," but "scarecrow."

Is cancer curable? That question goes to the root of the matter. It may be affirmed with truth that when a cancer has reached an advanced stage it cannot be cured by any method known to modern science; and it is a corollary, as certain as the dawn which follows the sunrise, that if there be no cure known to science there is no cure known to ignorance. The man who makes a profession of curing cancers indiscriminately is a "quack" and a knave. But this does not mean that there is no hope for a person whose cancer is treated at an early stage—far from it. There is a very simple rule which, if always observed, might save many lives. The rule is this, "Seek medical advice at once, so soon as any unusual and unexplained swelling is discovered in any part of the body." The family doctor will, in nine cases out of ten, know exactly how to deal with the strange visitor. If he is in any doubt he will send the case to an operating surgeon of repute. He will not probably send it to a cancer specialist, and quite rightly. Cancer specialists are not known at general hospitals; and it may be taken for granted nowadays that any disease which is not made a specialty of at a thoroughly-equipped general hospital like Guy's, St. Bartholomew's, St. Thomas's, or the London, does not need to be made a specialty of in ordinary practice. For science, for practical skill, for honesty, the persons most to be relied on in cases of cancer, as in other cases, are practitioners of known character who are trusted and consulted by the medical profession. The proper way of obtaining the best services of such men is to approach them through the medium of the family doctor.

In saying this we are not influenced by a desire to conserve medical etiquette or the interests of medical practitioners. Where there is danger to life we attach no more importance to mere etiquette or to purely selfish interests than we do to a wind that blows a single apple from a tree, or a wavelet that carries a toy-boat to the other side of the village pond. But we know of our own certain knowledge, and from long personal experience, that in this country at any rate the best medical and surgical skill and the truest honesty are to be found among the regular and known practitioners of the medical and surgical arts. Quacks are knaves and thieves, whether qualified practitioners or otherwise. They live by untruthful though plausible professions; they grow rich on the apprehensions and terrors of their clients. When a person is suffering from a slight illness, which Nature is quite competent to deal with, he may betake himself to a quack if he pleases, or, if he prefers it, to the cook. But when any man or woman has a new growth of any kind, let no time be wasted. The proper course to take is at once to seek the guidance of the highly skilled practitioner and to follow his advice implicitly. The result of such a course will be that the disease when curable will be cured; when not curable its evil effects will be reduced to a minimum, and the patient will be placed in the best possible circumstances for the prolongation of life, and for making existence as painless and as comfortable as possible whilst it endures. In these days the best professors of the arts of medicine and surgery have a deep sense of their responsibility, not only in the treatment of the peculiar ills from which mankind suffer, but also in the direction of enlightening and instructing the public mind. The mission of the doctor is to cure physical ills where possible, and to make the public so understand them that they shall not unduly alarm the imagination or depress the spirits. He who drives away ghosts and expels nightmares is as much a friend to humanity as he who makes the corn to grow or relieves physical ills.

